# Integration of CTA in the Diagnostic Workup of New Onset Chest Pain in Clinical Practice

**DOI:** 10.1155/2019/2647079

**Published:** 2019-07-07

**Authors:** Nazario Carrabba, Martina Berteotti, Giulia Taborchi, Francesca Ciatti, Manlio Acquafresca, Mario Moroni, Angela Migliorini, Vittorio Miele, Niccolò Marchionni, Renato Valenti

**Affiliations:** ^1^Cardiovascular and Thoracic Department, Careggi Hospital, Florence, Italy; ^2^Radiology Department, Careggi Hospital, Florence, Italy

## Abstract

**Background:**

Recently, NICE guidelines recommend the use of computed tomographic angiography (CTA) as the first line of investigation for new onset chest pain. We sought to evaluate the impact of the integration of CTA in the diagnostic workup, as either a first- or second-line of investigation, in the clinical practice for patients presenting with new onset chest pain, with suspicion that it may be due to coronary artery disease (CAD).

**Method and Results:**

From 2014 to 2016, 208 outpatients (mean age 63.8 ± 12.7, 37% female) with an unknown CAD diagnosis were evaluated. About half (n=106, 51%) received usual testing care plus CTA as a second-line investigation (group A), while the other half (n=102, 49%) received CTA as a first-line investigation (group B). Care decisions and test interpretations were made by the attending physician. Obstructive CAD (O-CAD) was defined as >50% stenosis in the principal branch. As determined by CTA, the rates of CAD in group A vs. group B were the following (P=0.001): 31.1% vs. 27.4% for normal/minimal CAD; 42.5% vs. 63.7% for no O-CAD; and 26.4% vs. 8.8% with O-CAD. Based on a diagnostic result of no O-CAD, invasive angiography was cancelled in 42.6% (n=45) of group A patients, and additional functional tests were cancelled for the same reason in 63.7% (n=65) of group B patients, without adverse events at median 3-year. The average diagnostic cost for patients in our study was lower in group B (206 vs. 324.42 euro; P<0.0001).

**Conclusions:**

In clinical practice, CTA, as a first- or second-line investigation, most commonly detected no O-CAD in new onset chest pain patients, leading us to safely avoid unnecessary ICA or additional functional tests. The use of CTA as a first-line investigation also appears to be cost saving, but its cost-effectiveness remains to be demonstrated in larger studies.

## 1. Introduction

For patients with suspected coronary artery disease (CAD), in many centers, the routine noninvasive diagnostic approach consists of stress testing, with the applied test modality depending on pretest probability, patient characteristics, local availability, and expertise. The exercise ECG is the most widely available and least expensive functional test, but it has a modest diagnostic performance. Conversely, the negative predictive value and the ability to exclude CAD are high for computed tomographic angiography (CTA) [1, 2]. However, CTA has a tendency to overestimate both the angiographic and hemodynamic severity of CAD, producing results which may necessitate further functional testing or ICA. Because the prevalence of obstructive-CAD (O-CAD) is low in real-world populations with stable chest pain symptoms [3], CTA could be an efficient and cost-effective first-line test. Recently, the PROMISE [4] and SCOT-HEART [5] studies have suggested that an evaluation strategy based on coronary CTA improves diagnostic certainty, as well as the efficiency of triage to invasive catheterization. It also may reduce radiation exposure when compared with functional stress testing, with similar rates of cardiac events. Moreover, the EVINCI [6] trial supports the use of CTA for stable chest pain, highlighting better performance in comparison with other imaging strategies. Following the publication of these studies, the National Institute for Health and Care Excellence (NICE) [7] recommended CTA as the first-line investigation for all patients presenting with chest pain due to suspected CAD. In the present prospective registry, we sought to evaluate the impact of the integration of CTA as a first- or second-line investigation in the diagnostic workup for the evaluation of outpatients presenting with stable chest pain in our tertiary center.

## 2. Material and Methods

**Figure 1:**
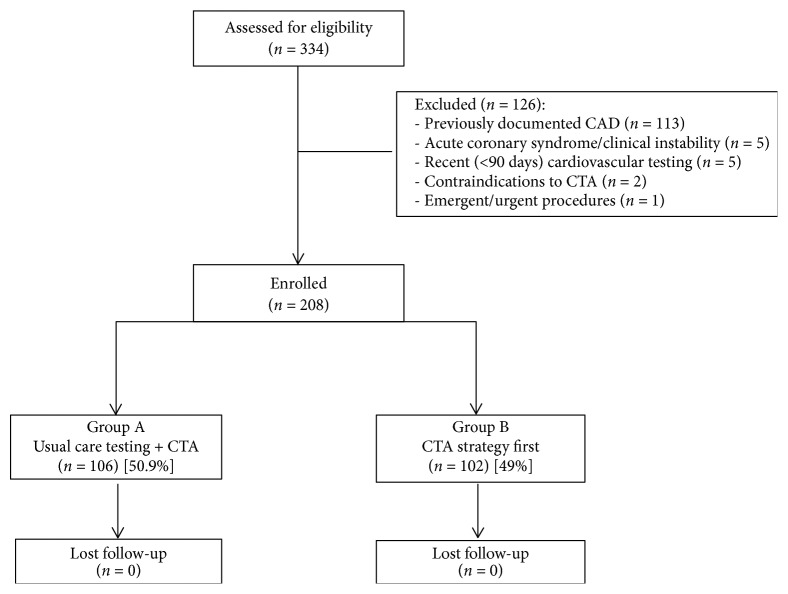
Study design flow diagram. CAD: coronary artery disease; CTA: computed tomographic angiography.

From May 2014 to February 2016, 334 consecutive symptomatic outpatients of age ≥18 years old, with new onset chest pain, were referred to our Cardiovascular and Thoracic Department of Careggi Hospital (Florence, Italy) for the suspicion of CAD. Exclusion criteria for the prospective registry were previously documented CAD (n=113), acute coronary syndrome or clinical instability (n=5), contraindications to CTA (n=2), and the need for emergent or urgent procedure (n=1). Additional exclusion criteria included recent cardiovascular testing (<90 days; n=5). Thus, 208 outpatients presenting with a low to intermediate likelihood of obstructive CAD were enrolled in this registry to evaluate the impact of integration of CTA in the diagnostic workup, using CTA as either a first- or second-line of investigation. Patients were enrolled by the attending physician, who, according to his behavior—and without any restriction—chose the patient's diagnostic workup, generating two groups of patients. Specifically, one group of patients receiving usual care testing (a symptom-limited exercise test, an echo stress test, or SPECT) according to the standard diagnostic workup, plus CTA as a second-line investigation, irrespective of the functional testing results, was classified as group A. Instead, patients receiving CTA as a first-line investigation were classified as group B (Figure 1). Care decisions and test interpretations were made by the attending physician, without any conditioning. All patients underwent CTA with a dual-source CTA scanner (52% with high-pitch mode, depending on the baseline heart rate), and followed scanning protocols satisfying Society of Cardiac Computed Tomography quality standards [8]. Normal/minimal CAD was defined as <30% stenosis in principal branches of the left or right coronary artery by visual estimate, while no O-CAD and O-CAD were defined as ≥30-50% and >50% stenosis, respectively. All subjects provided informed written consent, which was approved by the local ethics committee. At least 3 authors (N.C., M.B., and G.T.) had access to all data presented. All authors are responsible for the data integrity. Follow-up information for the occurrence of death, myocardial infarction, or hospitalization for coronary angioplasty was obtained by clinical visits or telephone interviews over the course of the following 3 years. Hospital records of all patients were screened for the occurrence of clinical events to confirm the obtained information.

### 2.1. CTA Scan Protocol

All CTAs were performed using a 128-slice dual-source CTA system (SOMATOM Definition Flash, Siemens Healthineers, Forchheim, Germany). The detector collimation was 2×64×0.6 mm, and a flying focal spot technique and a gantry rotation time of 280 msec were used. Both tubes were operated at 100 or 120 kV, depending on the body mass index of the patient. Scout-based automatic tube current modulation (Care Dose 4D, Siemens healthcare, Forchheim, Germany) was used, with the reference tube current-time product set at 320 mAs per rotation. The pitch was 3.4 for flash CTA mode. Oral and/or intravenous beta-blockers or oral ivabradine were administered if necessary, in an attempt to achieve a target heart rate <60 beats/min. All patients received sublingual nitrates. The contrast medium (Iomeron 400, Bracco Altana Pharma, Konstanz, Germany) and saline chaser were administered at 5 mL/s using a dual-head power injector (Empower, ACIST) into an antecubital vein, through an 18-gauge catheter. The patient's heart rate and ECG trace were recorded during examination. A test bolus scan was performed to determine the transit time. An injection of 15 mL of iodinated contrast medium was followed by a 30 mL saline chaser. The time until the peak opacification in the proximal ascending aorta was measured, and this time, plus 2 for standard protocol, or plus 5 for high-pitch protocol, was considered to represent the transit time of contrast agent. Sixty-five milliliters of contrast medium, followed by a 50 mL saline chaser, was administered, with bolus tracking using a region of interest (ROI) in the ascending aorta. The scan was automatically triggered when the tracking ROI reached a threshold of 100 Hounsfield units (HU) above baseline attenuation. In the flash mode (high-pitch spiral mode), prospective ECG-triggering was used to obtain a complete dataset in a single heartbeat starting at 60% of the R-R interval. In the sequential mode (Spiral technique), the centre of the data acquisition window was set at 70% of the R-R interval. The entire heart was covered in three or four heart-beats in a step-and-shoot fashion [9].

### 2.2. Image Reconstruction and Evaluation

Datasets for coronary arteries were reconstructed with a slice thickness of 0.6 mm, an increment of 0.4 mm, a field of view of 180 mm, a medium-soft convolution kernel (B26), and, in patients exhibiting coronary calcium, an additional sharp convolution kernel (B46). All reconstructed images were transferred to a dedicated workstation (MMWP, Siemens Healthcare, Forchheim, Germany). Axial images, multiplanar reformations, and maximum intensity projections were used to evaluate arteries. Coronary artery segments were classified according to a modified American Heart Association [10] protocol. Segments were evaluated if the luminal diameter met or exceeded 1.5 mm, as judged by two independent observers (N.C. and M.A., each with more than 9 years of coronary CTA experience). Any discordance in the interpretation was solved by a third observer (M.M. with more than 7 years reading experience). Image quality was assessed semiquantitatively using a four-point grading scale: (1) excellent (no artifacts, unrestricted evaluation), (2) good (minor artifacts, good diagnostic quality), (3) adequate (moderate artifacts, still acceptable and diagnostic), and (4) not assessable (severe artifacts impairing accurate evaluation). Images with a score of 1–3 were considered acceptable for diagnosis [8].

### 2.3. Radiation Dose Estimates

The radiation dose was reported as dose-length product (DLP) and effective dose (ED). For each patient, the ED was calculated using the formula DLP x 0.014, using the 0.014 conversion factor for chest radiation (in mSv/Gy/cm) [11].

### 2.4. Statistical Analysis

All variables are expressed as mean value ± SD or medians with interquartile ranges (25th to 75th percentile). Differences in patient characteristics, radiation dose, and image quality were compared using an independent-sample* t*-test (if normally distributed) or Mann-Whitney U-test (if not) for continuous variables, using *χ*^2^ or Fisher's exact test for categorical variables and the Kruskal-Wallis test for nonparametric data, as appropriate. The interrater agreement between the two observers in assessing image quality was calculated using Cohen's kappa statistics. Kappa results were interpreted as being either poor (*κ* < 0.20), fair (*κ*=0.21–0.40), moderate (*κ*=0.41–0.60), good (*κ*=0.61–0.80), very good (*κ*=0.81–0.90), or excellent (*κ*≥0.91). For economic analysis, unadjusted diagnostic costs were compared between the diagnostic strategies deployed by groups A and B using the nonparametric Wilcoxon rank sum test on all patients. For this economic analysis, the cost for the exercise test is 113.5 euros, the echo stress test is 181 euros, SPECT is 433 euros, and the CT scan is 206 euros. Statistical analyses were performed using commercially available software (SPSS, version 19.0, Chicago, IL, USA). A two-sided p-value of less than 0.05 was considered statistically significant.

## 3. Results

**Figure 2:**
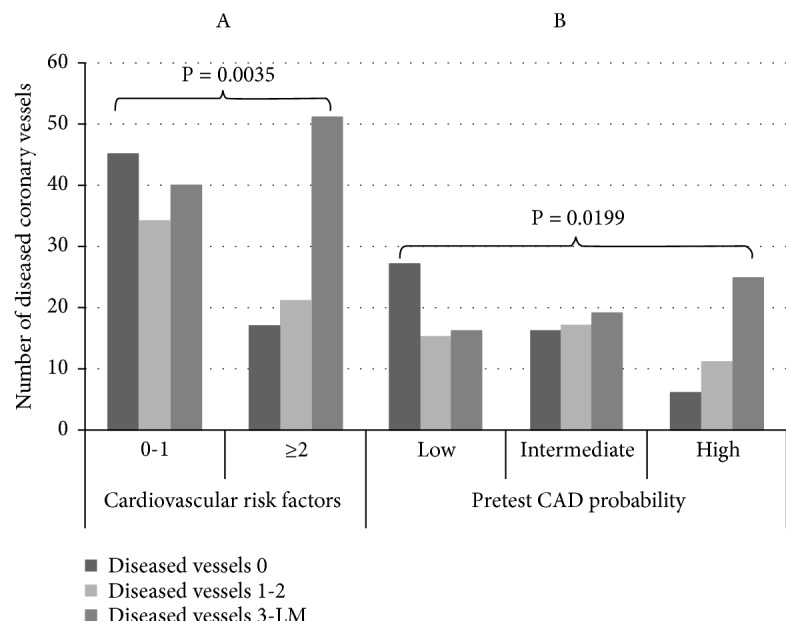
A: relationship between number of diseased coronary vessels and coronary risk factor (RF) ≥2 vs. RF 0-1 (P=0.0035). B: relationship between number of diseased coronary vessels and pretest probability low vs. intermediate and high (P=0.0199).

**Figure 3:**
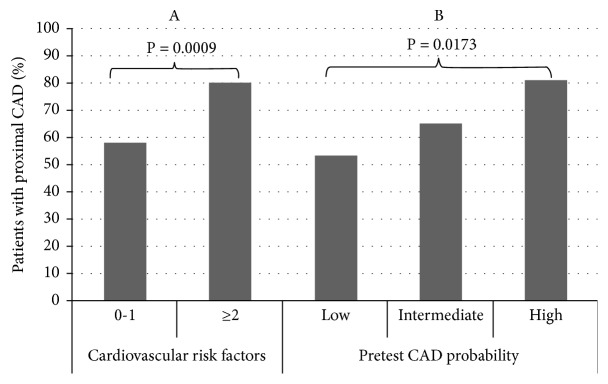
A: relationship between percentage of patients with proximal plaques and coronary risk factor (RF) ≥2 vs. RF 0-1 (P=0.0009). B: relationship between percentage of patients with proximal plaques and pretest probability low vs. intermediate and high (P=0.0173).

The 208 patients in the registry averaged 63.8 ± 12.7 years of age, and 37% were women. The clinical characteristics of the patients were similar, but group B (n=102, 49%) was older and the rate of female gender was higher in comparison with group A (n=106, 51%). All patients showed an intermediate risk profile (see Table 1). Among the patients in group A, 102 (96%) underwent symptom-limited exercise electrocardiography with the standard Bruce protocol, 3 (3%) underwent an echo stress test, and 1 (1%) was tested by SPECT. According to the results of the usual care strategy, group A patients were classified as exhibiting a negative (n=54, 50.9%), inconclusive (n=44, 41.5%), or positive (n=8, 7.5%) result of the functional test. Based on the results of CTA, as per patient analysis, the overall rate of patients with no O-CAD (52.8%) was the highest when compared to the rates of minimal/normal coronary artery (29.3%) and O-CAD (17.7%). Specifically, in group A, the percentage of normal/minimal lesion of coronary artery was 31.1% vs. 27.4% in group B; the percentage of no significant O-CAD was 42.5% in group A vs. 63.7% in group B; and the percentage of O-CAD was 26.4% in group A vs. 8.8% in group B (P=0.001) (Table 2). Per vessel analysis, the number of diseased coronary vessels was not significantly different in group A vs. group B patients (Table 2). Moreover, in patients with coronary risk factor (RF) ≥2 compared with patients with a coronary RF of 0-1, the number of diseased coronary vessels (P=0.0035) (Figure 2A), as well as the number of patients with proximal plaques (P=0.0009) (Figure 3A), was higher. Additionally, the number of diseased coronary vessels (P=0.0199) (Figure 2B), as well as the number of patients with proximal plaques (P=0.0173) (Figure 3B), was higher in patients with an intermediate or high tertile risk profile in comparison to patients with a low tertile risk. Per segment analysis, in group B patients, when compared with group A patients, the number of segments showing no obstructive plaques was higher (P=0.0034), whereas the number of segments showing obstructive plaques was lower, and the number of segments with no or minimal lesion was not different between the two groups (Table 2). Per plaque analysis, the noncalcified plaques were more frequently observed in group B patients with respect to group A patients, without difference in calcified or partially calcified plaques (Table 2). In group A patients, CTA, respectively, demonstrated 13 (24%), 10 (22.7%), and 5 (62.5%) patients with O-CAD, among patients whose results showed negative, inconclusive, and positive functional tests (see Table 3). After examining the results of CTA, the planned investigations changed mainly due to the exclusion or identification of O-CAD. Specifically, among group A patients, the 42.5% identified with no O-CAD had ICA cancelled, and 31.1% with normal/minimal CAD did not undergo other tests. Among group B patients, the 63.7% with no O-CAD avoided functional testing, and the 27.4% with normal/minimal CAD did not undergo other tests. The 26.4% and the 8.8% of patients with O-CAD among groups A and B, respectively, underwent ICA directly without additional noninvasive functional tests, considering the availability in catheterization lab of IVUS and FFR in case of the persistence of uncertainty of the significance of coronary stenosis after ICA evaluation. Overall, in patients presenting with O-CAD by CTA (n=37), ICA confirmed obstructive CAD in 92% (23/25) of cases. In 2 patients, the intravascular ultrasound did not confirm a case of O-CAD detected by CTA, demonstrating an overestimation of the degree of the coronary stenosis (sensitivity 100%, specificity 60%, positive predictive value 92% [95% CI 75.3 to 97.77], negative predictive value 100% [95% CI 43.85 to 99.99%], and accuracy 92.85%). In the remaining 12 patients, ICA was not performed due to plaques located in distal part of coronary vessel in 5 patients (3 from group A) and in minor branches in 7 patients (3 from group A). These patients with stable O-CAD were treated with optimal medical therapy. In case of the persistence of symptoms despite medical treatment, a PCI was performed 3 months after CTA, after the demonstration of large amount of myocardial ischemia (n=3) or positive fractional flow reserve (n=1).

### 3.1. Cost Analysis

The cost analysis revealed that the average diagnostic cost per patient for patients in group A was 324.42 euros (95% CI: 310.34-338.51); for patients in group B, it was 206 euros (P<0.0001). Thus, the diagnostic cost-savings was a difference of 118.42 euros while using CTA as a first-line investigative strategy, as compared to the standard strategy.

### 3.2. High-Pitch vs. Standard CTA

The patient heart rate was lower in those who underwent high-pitch CTA in comparison to those who underwent standard CTA (55±4 bpm vs. 67±7 bpm, P<0.001). The mean cumulative radiation exposure was also lower in patients receiving high-pitch vs. standard CTA mode (1.48±1.36 mSv vs. 8.10±3.14 mSv, P<0.001). The agreement between the two observers in assessing image quality was excellent, with a kappa result of 0.93. The number of segments exhibiting coronary vessels not able to be evaluated (quality score=4) was low and similar in both high-pitch and standard CTA mode: 1.2% vs. 1.04% (P=0.634), respectively. Following CTA, an allergic reaction to the contrast medium agent occurred in 1 patient, who was successfully treated with cortisone and antihistamine agents.

### 3.3. Follow-Up

Throughout a median 3-year follow-up (group A 36.3±11.2 months and group B 36.7±11.4 months, P=0.798), death and myocardial infarction did not occur in any of the patients with no O-CAD and cancelled ICA (from group A) nor in those with a cancelled functional test (from group B). The unplanned evaluations in the emergency department included two cases of palpitations, one for each group, and two cases of acute chest pain, one for each group; both were discharged from the hospital uneventfully.

## 4. Discussion

Currently, a great debate occurs about the functional and anatomic diagnostic evaluation of CAD in patients presenting with new onset chest pain. After the publication of recent studies [4–6], the NICE guidelines recommended the use of coronary CTA as a first-line diagnostic test evaluation for all patients presenting with new onset chest pain suspected for CAD [7]. Our findings confirm that in clinical practice the use of coronary CTA, given its strong negative predictive value [12], allows us to avoid unnecessary functional tests or a more expensive and potentially harmful ICA in patients showing normal coronary arteries or no O-CAD. Undoubtedly, CTA increased the identification of both obstructive and no O-CAD. While currently there is no clear guidance on how to manage the case of no O-CAD, the most common pattern of atherosclerosis observed in new onset chest pain patients, there is growing evidence of the beneficial effects of statin therapy [13]. Not surprisingly, in our registry, the burden of diseased vessels and the rate of proximal plaques by CTA increased with the presence of more than two coronary risk factors, as well as with the increase of tertile risk factor among all patients at intermediate-risk profile. Categorizing CAD on the basis of stenosis severity alone fails to account for the continuum of risk associated with no obstructive atherosclerotic plaques [14]. It will be interesting to see whether the identification of vulnerable plaques by CTA will improve the risk stratification of future cardiac events, beyond the risk profile of patients at presentation [15]. In symptomatic patients with a suspicion of CAD, the recurrence of chest pain is frequent and usually patients undergo an additional functional test, due to the uncertainty of CAD presence, or undergo ICA directly, according to the behavior of the attending physician and the availability of local facilities and expertise. Thus, not surprisingly, in the USA [16], as well as in a European study [17], 40% of ICA patients present no O-CAD, contributing to an inappropriate and unjustified consumption of financial resources. Currently, in patients with stable CAD, the rate of occurrence of events during a follow-up appears to be reduced, in comparison with that reported in Courage ERA [18] and, in a more recent study [3], it was as low as 1.5%. Moreover, for symptomatic patients with normal coronary arteries, no additional evaluation test is necessary until 5 years from CTA according to the large CONFIRM study [19]. In our registry, no events were reported during the median 3-year follow-up for patients showing normal coronary arteries as well as no O-CAD. More recently, the 5-year clinical outcome of SCOT-HEART trial [20] showed that the use of CTA versus standard care alone is associated with a lower subsequent risk of death from CAD or nonfatal myocardial infarction (2.3% vs. 3.9%; HR 0.59). This benefit was achieved without a greater long-term use of ICA and coronary revascularization in the CTA group. According to the design of the study, the SCOT-HEART trial encouraged the secondary prevention strategy, i.e., statin agents, in patients with no O-CAD. This strategy may be very important, considering that nearly half of subsequent myocardial infarctions occurred among patients with no O-CAD. Finally, in patients showing O-CAD involving small coronary vessels or marginal branches, we avoided performing ICA and angioplasty. These patients remained uneventful throughout the follow-up. In fact, in this setting, the advantage of angioplasty in comparison to optimal medical therapy remains to be demonstrated [21, 22]. Thus, it is conceivable that, in the near future, the role of CTA as a prevention strategy could be expanded, thus avoiding invasive evaluation for patients with minimal or no O-CAD, as well as promoting preventive therapies, and finally, improving the appropriateness of ICA—avoiding coronary angioplasty in small vessels or minor branches showing low-risk obstructive plaques.

### 4.1. Radiation Dose

The radiation dose remains the main concern regarding the use of CTA in clinical practice, due to the cancer risk it poses. According to ALARA dogma [11], all techniques should be improved in order to reduce the effective radiation dose. Our findings demonstrated a 6-fold reduction of radiation dose by using high-pitch mode with respect to standard CTA mode. However, the feasibility of the high-pitch CTA mode depends on the patient heart rate. Despite the aggressive heart rate strategy control with beta-blocker/ivabradine adopted in our centre, the high-pitch CTA mode was feasible in only half of the patients, underlying the difficulty in implementing the high-pitch CTA mode in clinical practice. It is important to note, however, that the radiation dose for nuclear stress testing was even higher, with an average level of 14 mSv, as noted in the literature, in comparison to that of the standard CTA scan [23]. In addition, the diagnostic certainty of chest pain may be lost, and the ICA examination may be necessary in the case of doubts raised by the results of functional tests. Finally, the lessening of the radiation dose obtained by this technique may affect the quality of the imaging. However, we note that in our registry, the reduction of effective radiation exposure obtained by high-pitch CTA mode did not have a significant impact on the imaging quality.

### 4.2. Limitations

This study was a single-center, observational registry with inherent limitations. First, due to the small sample size, caution is needed in the interpretation of the results. Second, the appropriateness of extrapolation of our findings to other centers will depend on the comparability of the clinical setting in terms of current diagnostic care, available technology, cost-accounting systems, and therapeutic management attitudes. Third, for the evaluation of outpatients with new onset chest pain, the combination of both anatomic and functional data could be the best diagnostic strategy [3]; however, the fractional flow reserve-CTA and the fusion/hybrid imaging is not available in our centre. Finally, in the present registry, the diagnostic CTA strategy is deemed to be cost-saving; however, the cost-effectiveness of CTA and the downstream health-care resource utilization both need to be evaluated in larger studies, though the NICE cost utility analysis of CTA appears favorable [7]. In addition, an important limitation of this registry is represented by the cost analysis performed by considering only the diagnostic workflow tests, whereas the cost saving related to the downstream ICA cancellation and avoided functional tests, and the additional costs related to the management of O-CAD were not factored into the evaluation. Despite these limitations, we included outpatients who were representative of those referred to the cardiology clinic for assessment of suspected angina due to CAD. A similar rate of O-CAD was found in our chest pain population as in the larger SCOT-HEART study (17.8% vs. 24%), and a similar majority of our patients underwent an exercise test as those reported in the SCOT-HEART study (96% vs. 85%), confirming this overall picture. In our study, 42% of the exercise tests were inconclusive and 24% of patients with negative stress tests had obstructive CAD. Only 62.5% of positive stress tests had obstructive CAD, confirming the moderate diagnostic value of the exercise test. Yet, considering the well-known overestimation of the prevalence of O-CAD based on the risk models [19] due to the low rate of O-CAD in real world populations with stable chest pain symptoms, CTA could be an efficient first-line test [4, 5, 7]. Thus, one should realize that in real-world setting, the use of CTA scan as a first-line diagnostic strategy for outpatients with new onset chest pain may be both feasible, safe, and cost-saving. However, a forward-looking economic investment strategy is necessary for the implementation and integration of CTA in the diagnostic workup for chest pain in the clinical practice [24].

### 4.3. Conclusions

Our registry confirms that in new onset chest pain patients no O-CAD was the pattern of atherosclerosis most commonly detected by the CTA, as first- or second-line investigation test. Implementation of CTA for clinical decision-making may influence the downstream diagnostic workflow of patients in real-world setting, leading us to safely avoid unnecessary ICA or additional functional tests. Although CTA appears to be cost-saving, the cost-effectiveness of CTA integration in the diagnostic workflow for suspicion of CAD in real world remains to be demonstrated in larger studies.

## Figures and Tables

**Table 1:** Baseline clinical characteristics of the study population.

	All	Group A	Group B	p-value
	(n=208)	(n= 106)	(n= 102)	
Mean age (years)	63.8±12.7	61.5±12.5	66.17±12.7	0.008
Male gender, n (%)	131 (63%)	77 (72.7%)	54 (52.9%)	0.004
Obesity, n (%)	8 (3.8%)	3 (2.8%)	5 (4.9%)	0.492
Hypertension, n (%)	114 (54.8%)	54 (50.9%)	60 (58.9%)	0.267
Hypercholesterolemia, n (%)	67 (32.2%)	39 (36.8%)	28 (27.4%)	0.181
Diabetes mellitus, n (%)	33 (15.9%)	21 (19.8%)	12 (11.8%)	0.130
Smoking habits, n (%)	45 (21.6%)	18 (17.0%)	27 (26.4%)	0.129
Family history of CAD, n (%)	57 (27.4%)	31 (29.2%)	26 (25.5%)	0.641
Pretest probability	55.97±30.92	55.33±22.05	56.73±21.42	0.692

Data are presented as the mean±SD or as number (percentage) of patients; CAD: coronary artery disease; hypercholesterolemia: total cholesterol >5 mmol/L, low-density lipoprotein >3 mmol/L, or on lipid-lowering medication.

**Table 2:** CTA angiographic characteristics by groups.

	Group A	Group B	p-value
	(n = 106)	(n = 102)	
*Analysis by patient*			
No lesions or minimal lesions, n (%)	33 (31.1)	28 (27.4)	0.001
No obstructive lesions, n (%)	45 (42.6)	65 (63.7)	
Obstructive lesions, n (%)	28 (26.4)	9 (8.8)	
1 vessel, n (%)	10 (9.4)	18 (17.6)	0.369
2 vessels, n (%)	15 (14.1)	14 (13.7)	
3 vessels, n (%)	11 (10.4)	8 (7.8)	
Left main, n (%)	38 (35.8)	34 (33.3)	

*Analysis by segment*			
No lesions or minimal lesions, median (IQR)	0 (0-0)	0 (0-0)	0.164
Non obstructive lesions, median (IQR)	0 (0-3)	2 (0-4)	0.003
Obstructive lesions, median (IQR)	0 (0-1)	0 (0-0)	0.001

*Analysis by plaque*			
Noncalcified plaques, median (IQR)	0 (0-0)	0 (0-0)	0.015
Partially calcified plaques, median (IQR)	0 (0-3)	1 (0-2)	0.870
Calcified plaques, median (IQR)	0 (0-1)	0 (0-1)	0.296

Data are presented as number (%) or median (IQR: interquartile range).

**Table 3:** Functional test and CTA results of Group A and Group B patients.

Functional test results		CTA results		
		Obstructive CAD	No obstructive CAD	Minimal lesions/ no CAD
*Group A (n = 106)*				

All, n (%)		28 (26.4%)	45 (42.5%)	33 (31.1%)
Negative, n (%)	54 (50.9%)	13 (24.1%)	23 (42.6%)	18 (33.3%)
Positive, n (%)	8 (7.5%)	5 (62.5%)	1 (12.5%)	2 (25.0%)
Inconclusive, n (%)	44 (41.5%)	10 (22.7%)	21 (47.7%)	13 (29.5%)

* Group B (n = 102)*				

All, n (%)		9 (8.8%)	65 (63.7%)	28 (27.4%)
Negative, n (%)	-	-	-	-
Positive, n (%)	-	-	-	-
Inconclusive, n (%)	-	-	-	-

Data are presented as number (percentage).

## Data Availability

The data used to support the findings of this study are available from the corresponding author upon request.
